# Comparison of frictional resistance between self-ligating and conventional brackets tied with elastomeric and metal ligature in orthodontic archwires

**DOI:** 10.1590/2176-9451.19.3.114-119.oar

**Published:** 2014

**Authors:** Vanessa Vieira Leite, Murilo Baena Lopes, Alcides Gonini Júnior, Marcio Rodrigues de Almeida, Sandra Kiss Moura, Renato Rodrigues de Almeida

**Affiliations:** 1 MSc in Orthodontics, Northen University of Paraná (UNOPAR).; 2 Professor, Northen University of Paraná (UNOPAR).; 3 Adjunct professor, Northen University of Paraná (UNOPAR).; 4 Full professor, School of Dentistry - University of São Paulo/Bauru Professor, Northen University of Paraná (UNOPAR).

**Keywords:** Dental brackets, Wires, Friction, Self-ligating

## Abstract

**Objective:**

To compare the frictional resistance between self-ligating and conventional brackets tied to different types of wire.

**Material and Methods:**

Abzil Kirium Capelozza (Pattern I) and Easy Clip (Roth prescription) incisor brackets were used. An elastomeric ligature or a ligating wire 0.10-in was used to ligate the wire to the Abzil bracket. Three types of orthodontic archwire alloys were assessed: 0.016-in NiTi wire, 0.016 x 0.021-in NiTi wire and 0.019 x 0.025-in steel wire. Ten observations were carried out for each bracket-archwire angulation combination. Brackets were mounted in a special appliance, positioned at 90 degrees in relation to the wire and tested in two angulations. Frictional test was performed in a Universal Testing Machine at 5 mm/min and 10 mm of displacement. The means (MPa) were submitted to ANOVA and Tukey's test set at 5% of significance. The surfaces of wires and brackets were observed at SEM.

**Results:**

Steel-tied brackets (16.48 ± 8.31) showed higher means of frictional resistance than elastomeric-tied brackets (4.29 ± 2.16 ) and self-ligating brackets (1.66 ± 1.57) (P < 0.05), which also differed from each other (P < 0.05). As for the type of wire, 0.019 x 0.025-in steel wire (5.67 ± 3.97) showed lower means (P < 0.05) than 0.16-in NiTi wire (8.26 ± 10.92) and 0.016 x 0.021-in NiTi wire (8.51 ± 7.95), which did not differ from each other (P > 0.05). No statistical differences (P > 0.05) were found between zero (7.76 ± 8.46) and five-degree (7.19 ± 7.93) angulations.

**Conclusions:**

Friction was influenced not only by the type of bracket, but also by the ligating systems. Different morphological aspects were observed for the brackets and wires studied

## Abstract (pt)

**Objetivo:**

comparar a resistência ao atrito entre braquetes autoligáveis e braquetes convencionais, variando o tipo de fio.

**Métodos:**

foram usados braquetes incisivos Abzil Kirium Capelozza e Easy Clip. Uma ligadura elastomérica ou um fio ligável de 0,010" foi usado para ligação do fio no braquete Abzil. Três tipos de ligas de fio ortodôntico foram avaliadas: fio de NiTi de 0,016"; fio de NiTi de 0,016" x 0,022"; e fio de aço de 0,019" x 0,025". Dez observações foram feitas para cada combinação "braquete-fio-inclinação". Os braquetes foram montados em um aparelho especial, posicionados a 90° em relação ao fio, e testados em duas inclinações. O teste de atrito foi realizado na máquina universal de ensaios, a 5mm/min e com 10mm de deslocamento. As médias (em MPa) foram submetidas aos testes ANOVA e de Tukey, a 5% de significância. As superfícies dos fios e dos braquetes foram observadas no MEV.

**Resultados:**

o braquete com amarrilhos metálicos (16,48 ± 8,31MPa) apresentou médias mais altas que o braquete com ligaduras elastoméricas (4,29 ± 2,16MPa) e o braquete autoligável (1,66 ± 1,57) (p < 0,05), os quais também diferiram entre si (p < 0,05). Quanto ao tipo do fio, o fio de aço de 0,019" x 0,025" (5,67 ± 3,97MPa) apresentou médias mais baixas (p < 0,05) que os fios de NiTi de 0,016" (8,26 ± 10,92MPa) e de 0,016" x 0,022" (8,51 ± 7,95), os quais não diferiram entre si (p > 0,05). Nenhuma diferença estatística (p > 0,05) foi encontrada entre inclinações de zero (7,76 ± 8,46) e cinco (7,19 ± 7,93) graus.

**Conclusão:**

o atrito foi influenciado pelo tipo de braquete e sistemas de ligaduras. Diferentes aspectos morfológicos foram observados para os braquetes e fios estudados.

## INTRODUCTION

Orthodontic sliding mechanics is one of the most common methods of translating a tooth in a mesiodistal way, i.e., with canine or anterior retraction. In this technique, tooth movement can be accomplished by free body movement or by guidance of a tooth along an archwire.^2^ The major disadvantage of this last mechanism is friction generated between the bracket and the archwire, which tends to resist movement of the bracket and tooth in the desired direction.^2^

Friction is only one part which resistance to movement consists of when a bracket slides along an archwire.^4,9^ It is determined by the type and size of the archwire, type of bracket, angulation between the archwire and the bracket slot and the method of ligation. Since this force operates in the opposite direction of the mobile body, it is important that it be eliminated or minimized when orthodontic tooth movement is being planned,^2,8,27^ otherwise it may delay tooth movement, increase anchorage requirement, or both.^6,21^

Tooth movement can occur when applied forces adequately overcome friction at the bracket slot-archwire interface.^22^ Should a high level of frictional force between the bracket slot and the archwire occur, it might cause binding between the two components and result in little or no tooth movement.^14^

Several "friction-free" brackets, such as the self-ligating ones, have been recently developed.^6^ They consist of a preadjusted appliance with a mechanical device built into the bracket in order to close off the bracket slot.^14^ This ligatureless bracket system has the advantage of reducing chair time, promoting better oral hygiene and low frictional resistance due to better sliding mechanics.^14,15,26^ In addition, it may eliminate potential chances of soft tissue laceration caused by the use of stainless steel wires.^24^

Different experimental methods have demonstrated significant decrease in friction with self-ligating brackets in comparison to conventional ones.^14^ Thus, this article aims at assessing frictional resistance and the influence of angulation in the bracket-archwire interface by means of using different types of wires.

## MATERIAL AND METHODS

The following maxillary central incisor brackets (angulation 5º) were used in the study: Conventional preadjusted brackets with a slot of 0.022 x 0.030-in Abzil Kirium Capelozza prescription- pattern I- (3M Unitek, São José do Rio Preto, SP, Brazil), self-ligating brackets with a slot of 0.022 x 0.027-in (Roth prescription) (Aditek, Cravinhos, SP, Brazil). It is worth noting that the type of bracket and wires used for this study were made available from a previously published research.^4^ Furthermore, the orthodontic brackets assessed in the present study represent the brackets most widely used by clinicians nowadays.

An elastomeric ligature (3M Unitek) or a 0.10-in ligating wire was used for archwire ligation in the Abzil bracket. Three types of orthodontic archwire alloys were evaluated: 0.016-in NiTi wire, 0.016 x 0.021-in NiTi wire and 0.019 x 0.025-in stainless steel wire (Damon Universal, Ormco Corp., Orange, CA, USA). The brackets used on the right side had 0.022-in slots. Ten observations were carried out for each bracket-archwire combination. Each archwire sample was drawn only once through a bracket. Thus, 180 bracket-archwire readings were taken for the study.

**Figure 1:**
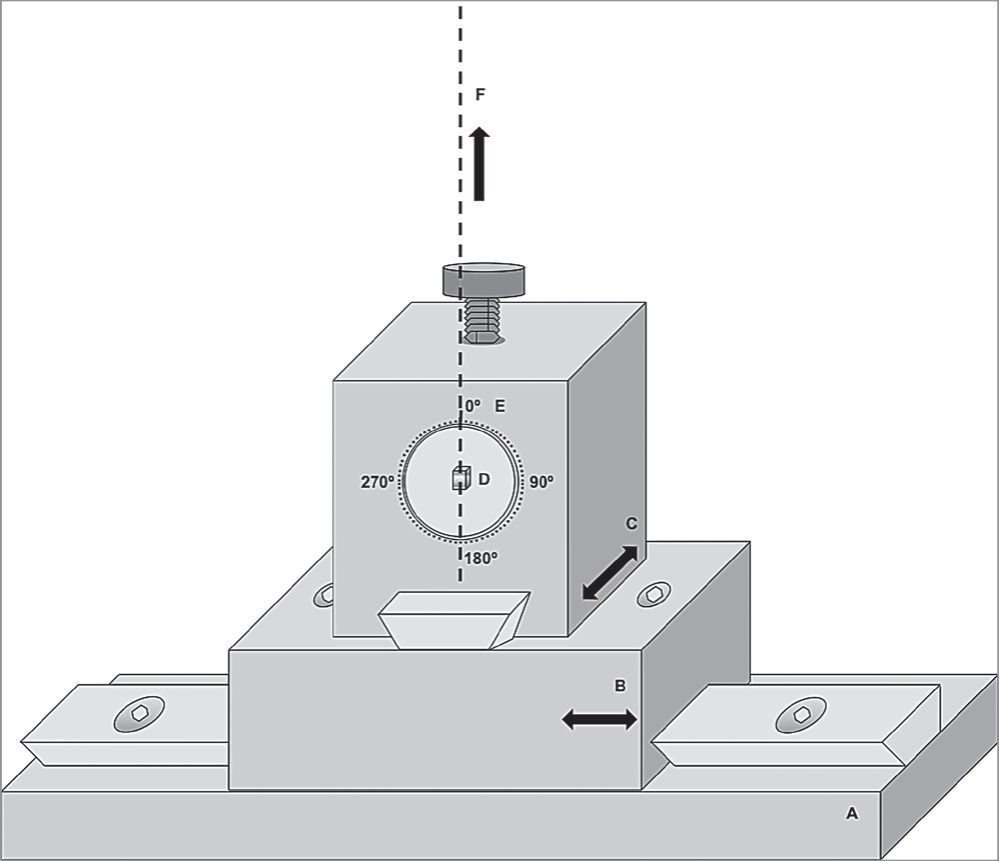
Testing appliance design: base locked to the testing machine (A), horizontal movable bases for bracket/wire alignment (B and C), bracket bonded to plastic pedestal (D), 360-degree display (E) and wire specimen attached to the load cell (F).

The frictional test was carried out in a special appliance (Fig 1) consisting of a base, in which the brackets bonded in the acrylic cylinder with cyanoacrylate adhesive (Superbonder gel, Loctite, Itapevi, SP, Brazil) were positioned at 90 degrees to the wire; and a mobile part where the wire was fixed. The appliance was mounted in a Universal Testing Machine (DL2000, Emic, São José dos Pinhais, PR, Brazil) and tested at 5 mm/min using 10 mm of displacement for all specimens^23^ at zero and five-degree angulations (Fig 1) with a load cell of 50 Kgf. The maximum values for each test were recorded. Means (MPa) were submitted to ANOVA and Tukey's test set at 5% of significance.

In addition, the surfaces of wires and brackets were observed at a Scanning Electron Microscope (SSX-550, Shimadzu, Tokyo, Japan) before and after testing.

## RESULTS

When the groups were analyzed according to the type of bracket, steel-tied brackets (16.48 ± 8.31) showed higher means, followed by elastomeric-tied brackets (4.29 ± 2.16) and self-ligating brackets (1.66 ± 1.57) All groups showed statistical differences (P < 0.05) when compared to each other (Table 1). When the type of wire was analyzed, 0.019 x 0.025-in stainless steel wire (5.67 ± 3.97) showed lower means, with statistical difference (P < 0.05) in comparison to 0.16-in NiTi wire (8.26±10.92) and 0.016 x 0.021-in NiTi wire (8.51 ± 7.95), which did not differ from each other (Table 2). When angulation was analyzed, no statistical differences (P > 0.05) were found between zero (7.76 ± 8.46) and five (7.19 ± 7.93) degrees (Table 3). The results of combined factors, type of bracket, type of wire and angulation are shown in Table 4.

**Table 1:** Means (MPa) and standard deviation of bracket type.

Steel-tied	16.48 ± 8.31^a^
Elastomeric-tied	4.29 ± 2.16^b^
Self-ligating	1.66 ± 1.57^c^

Different lower-case letters in the column indicate significative difference (P < 0.05)

**Table 2:** Means (MPa) and standard deviation of wire type.

0.016-in NiTi wire	8.26 ± 10.92a
0.016 x 0.021-in NiTi wire	8.51 ± 7.95^a^
0.019 x 0.025-in Stainless Steel wire	5.67 ± 3.97^b^

Different lower-case letters in the column indicate significant difference (P < 0.05)

**Table 3:** Means (MPa) and standard deviation of bracket angulation.

0°	7.76 ± 8.46a
5°	7.19 ± 7.93^a^

Different lower-case letters in the column indicate significant difference (P < 0.05)

**Table 4:** Means (MPa) and standard deviation of the type of bracket, wire and angulation.

	0.016-in NiTi wire		0.016 x 0.021-in NiTi wire		0.019 x 0.025-in stainless steel wire	
	0°	5°	0°	5°	0°	5°
Steel-tied	22.72 ± 9.28^bB^	19.10 ± 11.57^bB^	15.54 ± 5.33^abB^	21.28 ± 4.92^bB^	9.18 ± 2.32^aB^	11.05 ± 0.71^aB^
Elastomeric-tied	3.82 ± 1.83^aA^	3.77 ± 1.34^aA^	5.57 ± 1.73^aA^	4.03 ± 2.00^aA^	2.41 ± 0.57^aA^	6.15 ± 2.82^aAB^
Self-ligating	0.08 ± 0.08^aA^	0.06 ± 0.06^aA^	2.46 ± 0.94^aA^	2.17 ± 0.96^aA^	2.94 ± 1.87^aAB^	2.28 ± 1.39^aA^

According to Tukey's test, different lower-case letters in the column or different upper-case letter in the line referring to the same material indicate significant difference (P < 0.05).

**Figure 2:**
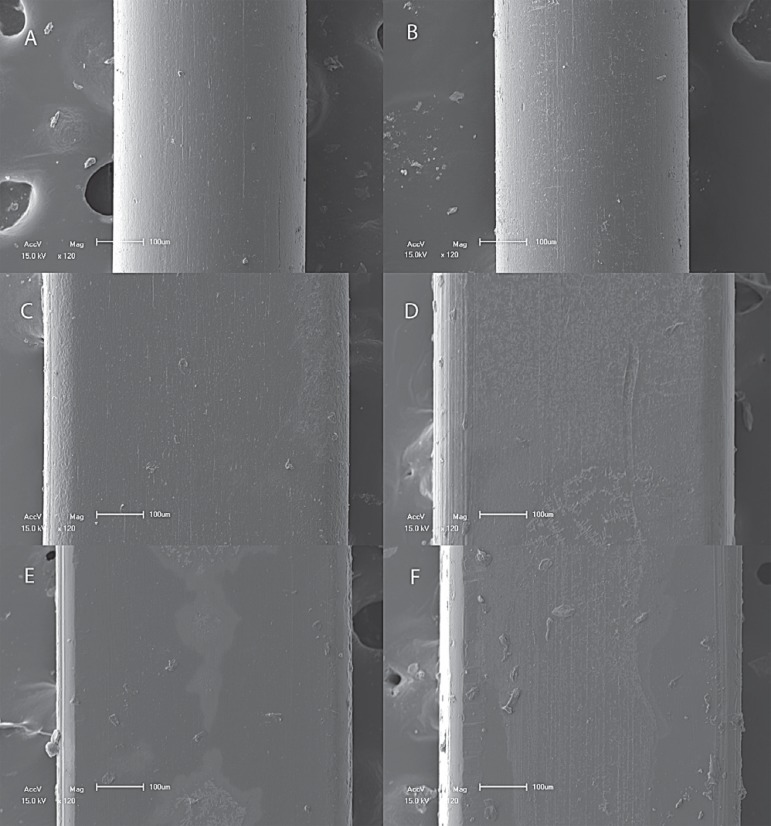
Wires before and after friction, respectively: 0.016-in NiTi wire (A and B), 0.016 x 0.021-in NiTi wire (C and D) and 0.019 x 0.025-in stainless steel wire (E and F).

**Figure 3:**
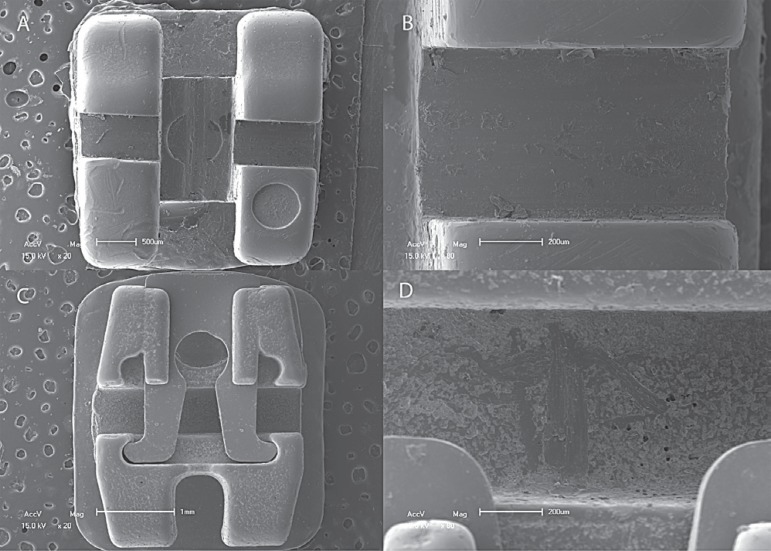
Steel and elastomeric-tied brackets (A), magnified slot (B); selfligating bracket (C), magnified slot (D).

Electronic microscopic analysis of wires showed a rougher surface for NiTi wires (Figs 2A and 2C) in comparison to stainless steel ones (Fig 2E) before sliding. The stainless steel wire seemed to have a smoother surface, but after sliding, it revealed wear tracks (Fig 2F). These tracks were more evident in Nitinol wires (Figs 2B and 2D) after sliding. Analysis of bracket surfaces showed a poorly polished surface for conventional (Figs 3A and 3B) and self-ligating ones (Figs 3C and 3D).

## DISCUSSION

To date, there are two different types of self-ligating brackets available: Active brackets, which feature a spring clip actively pressing against the archwire; and passive brackets, of which self-ligating clip does not press the wire.^23^ The present laboratory study was designed to compare friction produced by a passive self-ligating bracket and a conventional metal bracket with different wire dimensions and angulations.

Conventional ligation methods (stainless steel ligature wires or polymeric O-rings) apply force to the archwire pushing it against the depth of the slot, thus, increasing friction.^7^ Conversely, passive self-ligating brackets are characterized by the presence of a fourth mobile wall that converts the slot into a tube, allowing the wire to freely move inside the bracket slot, thus, reducing friction levels.^11^

This could be observed in the present study in which self-ligating brackets showed lower friction resistance in comparison to steel-tied and elastomeric-tied brackets, as demonstrated by Hain et al.^12^ Elastomeric-tied brackets showed lower friction in comparison to steel-tied ones, thereby corroborating the findings by Bazakidou et al.^1^ This would be due to the lack of tight contact, as shown by a steel or elastomeric bracket tied around the archwire.^24^ In clinical conditions, low friction may be important, whether in retracting a tooth along a continuous archwire or consolidating space.

With regard to wire alloy, stainless steel was found to be associated with significantly lower levels of frictional resistance in comparison to nickel-titanium alloy. This is in agreement with most reports found in the literature.^8,13,16,17,20^ When the morphological features of wires and brackets were analyzed by SEM, a more irregular surface was found in NiTi wires in comparison to steel wires before and after sliding. These findings may explain the friction values observed in the experimental conditions. Surface roughness may play a significant role in the amount of friction produced, as bracket design and ligation technique do.

Bowden and Tabor^3^ stated that friction was known to be largely determined by surface roughness. Similarly to the results of the present study, Pratten et al^20^ also observed lower frictional resistance for stainless steel brackets as a result of lower surface roughness and greater frictional resistance for NiTi arch wires. Stannard et al^25^ also microscopically examined different wires and found that, at first, stainless steel seemed to have a polished surface, however, it revealed wear tracks after sliding. Nitinol wire had a fibrous structure, which indicates lack of polishing during manufacture, and no observable wear tracks.

Elayyan et al^9^ speculated that surface quality of archwires might affect the area of surface contact, thereby modifying corrosion behavior and biocompatibility. According to their study, irregular surfaces microscopically observed might lead to plaque accumulation inside the surface defects, thus, affecting tooth movement due to entrapment of bracket edges inside them. Moreover, the contact area between the wire and the bracket surface can influence friction,^8,18^ which was not considered in the present study.

This study corroborates a previous study^19^ in which Nitinol and stainless steel arch wires were compared. The author reported lower friction for Nitinol only when wire-bracket angulation was > 5°. In the present experiment, no angulation higher than 5º was used and no statistical difference was found. According to Read-Ward,^21^ the effect of increasing angulation resulted in increased friction, which may be associated with the larger width of the wire when compared to the bracket width slot, producing a larger contact area and probably larger frictional forces.

The present study was not capable of simulating clinical conditions with all the attended variables. In future clinical studies, it would be of great interest to investigate whether self-ligating brackets actually result in faster and more efficient treatment, especially if aging alterations of the self-ligating brackets clip - occurring during the course of orthodontic treatment and which may modify the forces generated during wire engagement in comparison to elastomeric ligatures that are affected by moisture and heat - exhibit rapid force loss and are permanently deformed when stretched.

## CONCLUSION

1. Friction was influenced not only by the type of bracket, but also by the ligation system. Self-ligating brackets showed lower means.
2. There were no differences between zero and five-degree angulations with regard to frictional resistance
